# Pomalidomide, bortezomib and low-dose dexamethasone in lenalidomide-refractory and proteasome inhibitor-exposed myeloma

**DOI:** 10.1038/leu.2017.173

**Published:** 2017-06-23

**Authors:** P G Richardson, C C Hofmeister, N S Raje, D S Siegel, S Lonial, J Laubach, Y A Efebera, D H Vesole, A K Nooka, J Rosenblatt, D Doss, M H Zaki, A Bensmaine, J Herring, Y Li, L Watkins, M S Chen, K C Anderson

**Affiliations:** 1 Jerome Lipper Multiple Myeloma Center, Department of Medical Oncology, Dana-Farber Cancer Institute, Harvard Medical School, Boston, MA, USA; 2 Division of Hematology, Department of Internal Medicine, The Ohio State University, Columbus, OH, USA; 3 Massachusetts General Hospital, Boston, MA, USA; 4 John Theurer Cancer Center, Hackensack University Medical Center, Hackensack, NJ, USA; 5 Department of Hematology and Medical Oncology, Winship Cancer Institute, Emory University School of Medicine, Atlanta, GA, USA; 6 Beth Israel Deaconess Medical Center, Harvard Medical School, Boston, MA, USA; 7 Celgene Corporation, Summit, NJ, USA

## Abstract

This phase 1 dose-escalation study evaluated pomalidomide, bortezomib (subcutaneous (SC) or intravenous (IV)) and low-dose dexamethasone (LoDEX) in lenalidomide-refractory and proteasome inhibitor-exposed relapsed or relapsed and refractory multiple myeloma (RRMM). In 21-day cycles, patients received pomalidomide (1–4 mg days 1–14), bortezomib (1–1.3 mg/m^2^ days 1, 4, 8 and 11 for cycles 1–8; days 1 and 8 for cycle ⩾9) and LoDEX. Primary endpoint was to determine the maximum tolerated dose (MTD). Thirty-four patients enrolled: 12 during escalation, 10 in the MTD IV bortezomib cohort and 12 in the MTD SC bortezomib cohort. Patients received a median of 2 prior lines of therapy; 97% bortezomib exposed. With no dose-limiting toxicities, MTD was defined as the maximum planned dose: pomalidomide 4 mg, bortezomib 1.3 mg/m^2^ and LoDEX. All patients discontinued treatment by data cutoff (2 April 2015). The most common grade 3/4 treatment-emergent adverse events were neutropenia (44%) and thrombocytopenia (26%), which occurred more frequently with IV than SC bortezomib. No grade 3/4 peripheral neuropathy or deep vein thrombosis was reported. Overall response rate was 65%. Median duration of response was 7.4 months. Pomalidomide, bortezomib and LoDEX was well tolerated and effective in lenalidomide-refractory and bortezomib-exposed patients with RRMM.

## Introduction

The introduction of immunomodulatory agents and proteasome inhibitors (PIs) has transformed multiple myeloma (MM) therapy over the past decades, with significant improvement in response rates, progression-free survival (PFS) and overall survival (OS).^1, 2^ However, relapse is inevitable in almost all patients, and recurrence of MM is typically more aggressive with each subsequent relapse, justifying the development of new combinations mainly in MM-refractory disease.^3^

In preclinical studies, the immunomodulatory agents thalidomide and lenalidomide were each shown to potentiate the activity of bortezomib in combination with dexamethasone.^4, 5^ Results from clinical studies confirmed the antimyeloma activity of the combination of lenalidomide and bortezomib in patients with MM.^6, 7, 8^ The phase 3 SWOG S0777 trial demonstrated a significantly longer PFS (median of 43 vs 30 months; two-sided *P*=0.0037) and OS (median of 75 vs 64 months; two-sided *P*=0.025) with the triplet combination of lenalidomide, bortezomib and dexamethasone compared with lenalidomide and dexamethasone in patients with newly diagnosed MM.^8^ Similarly in patients with relapsed or relapsed and refractory MM (RRMM), the combination of lenalidomide, bortezomib and low-dose dexamethasone (LoDEX) has displayed antimyeloma activity, with an overall response rate (ORR) of 64% and a 6-month PFS rate of 75%.^7^ More than half of the patients in the trial had prior exposure to bortezomib, and three-quarters had prior exposure to thalidomide, indicating that the combination of lenalidomide and bortezomib could achieve responses in patients previously exposed to thalidomide and/or bortezomib.^7^

Pomalidomide is an oral IMiD immunomodulatory agent with significant activity in RRMM.^9^ With direct tumoricidal, potent immune-activating, antiangiogenic and anti-inflammatory activities, pomalidomide is being extensively investigated in combination with other agents that have complementary mechanisms of action.

The approved treatment schedule of pomalidomide is 4 mg/day on days 1–21 of a 28-day cycle, given in combination with LoDEX. The regimen is approved in the United States for patients with MM who have received ⩾2 prior therapies including lenalidomide and a PI and have had disease progression within 60 days of completing their last therapy.^10, 11, 12, 13^ In patients with RRMM who were heavily pretreated with lenalidomide and bortezomib, treatment with pomalidomide and LoDEX delayed disease progression (median PFS, 4.0 vs 1.9 months; hazard ratio, 0.48; *P*<0.0001) and extended survival (median OS, 12.7 vs 8.1 months; hazard ratio, 0.74; *P*=0.0285) compared with high-dose dexamethasone.^11, 12, 13^ The efficacy and tolerability of pomalidomide were maintained in all subgroups, including the elderly,^14^ patients with high-risk cytogenetics^15, 16^ and those with moderate renal impairment.^17, 18^

The current study was a prospective phase 1, multicenter, dose-escalation trial of pomalidomide, bortezomib and LoDEX in lenalidomide-refractory and PI-exposed patients. During the trial in 2012, subcutaneous (SC) bortezomib was approved by the US Food and Drug Administration based on its noninferiority to intravenous (IV) bortezomib and improved safety profile in RRMM.^19^ Therefore, an additional cohort of patients receiving pomalidomide, SC bortezomib and LoDEX was included; however, this study was not designed to compare SC vs IV bortezomib. We report the results of patients treated with pomalidomide, IV bortezomib and LoDEX in the escalation cohorts, the maximum tolerated dose (MTD) plus IV bortezomib cohort, the MTD plus SC bortezomib cohort and the entire study population.

## Materials and Methods

### Study participants

This phase 1, open-label, dose-escalation study (MM-005; ClinicalTrials.gov NCT01497093) was conducted in 6 centers in the United States. Patients enrolled into the study were aged ⩾18 years with an Eastern Cooperative Oncology Group performance status of ⩽2 and were required to have a documented diagnosis of MM with measurable disease by serum (⩾0.5 g/dl) and/or urine protein (⩾200 mg/24 h) electrophoresis. All patients must have received 1–4 prior antimyeloma therapies. Prior treatment must have included ⩾2 consecutive cycles of lenalidomide and ⩾2 consecutive cycles of a PI. Patients were required to be refractory to the last lenalidomide-containing regimen but were not allowed to be refractory to bortezomib <1.3 mg/m^2^ twice weekly either as a single agent or in combination. Refractory status/disease was defined as documented disease progression during treatment or within 60 days after the last dose of either agent given as monotherapy or with combination treatment.

Patients were ineligible if they had previously received pomalidomide; had hypersensitivity to thalidomide, lenalidomide, bortezomib or dexamethasone; or had grade ⩾3 rash during prior therapy with thalidomide or lenalidomide. Patients with grade ⩾2 peripheral neuropathy or a history of congestive heart failure, myocardial infarction within 12 months of starting the study or unstable or poorly controlled angina pectoris were excluded. Patients with any of the following laboratory abnormalities were also ineligible for study participation: absolute neutrophil count of <1000/μl; platelet count of <75 000/μl for patients in whom <50% of bone marrow nucleated cells were plasma cells or <30 000/μl for patients in whom ⩾50% of bone marrow cellularity were plasma cells; creatinine clearance of <45 ml/min according to the Cockcroft-Gault formula or on collection of 24-h urine; hemoglobin of <8 g/dl; corrected serum calcium of >3.4 mmol/l; total bilirubin level of >1.5 × upper limit of normal; or liver enzyme levels of >3 × upper limit of normal. Additional exclusion criteria included other malignancies unless the patient was disease free for ⩾5 years or the malignancy included basal cell or squamous cell skin cancer, *in situ* cervical, breast or prostate cancer (T1a or T1b or otherwise considered curable); gastrointestinal disease that may interfere with pomalidomide absorption; plasmapheresis, major surgery, radiation therapy or any antimyeloma treatment for ⩽14 days of therapy; other conditions that require chronic steroids or immunosuppression; or known infection with human immunodeficiency virus or hepatitis B or C virus.

This study was approved by the institutional review board or independent ethics committee at each participating center before initiation of any study procedures and was conducted in accordance with the principles for Good Clinical Practice (as outlined by the International Conference on Harmonisation E6 requirements) and the Declaration of Helsinki. Before the start of the study, all patients provided written informed consent. All authors had access to the primary clinical trial data and, with the sponsor, analyzed and interpreted the data.

### Study design and treatment

**Figure 1:**
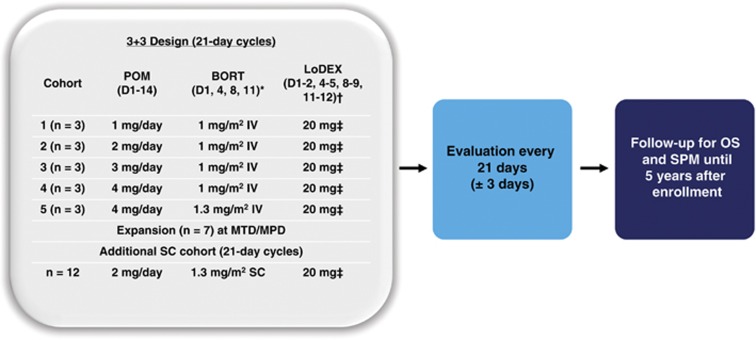
MM-005 trial design. MM-005 included 5 dose-escalation cohorts. Three patients were treated at each dose level, and 7 additional patients were treated at the MTD in the expansion phase of the trial. An additional cohort of patients treated with SC bortezomib (BORT) was included (*n*=12). Patients were evaluated every 21 days until treatment discontinuation. Patients will undergo long-term follow-up for OS and secondary primary malignancies (SPM). *For cycles 1–8, then D1 and D8 for cycle ⩾9. ^†^For cycles 1–8, then D1–2 and D8–9 for cycles ⩾9. ^‡^10 mg for patients aged >75 years. POM, pomalidomide.

This dose-escalation trial used a 3+3 design to determine the primary endpoint of MTD for the combination of pomalidomide, IV bortezomib and LoDEX in patients with RRMM. There were 5 dosing cohorts (Figure 1). Patients in cohort 5 received the maximum planned dose (MPD) of pomalidomide 4 mg, IV bortezomib 1.3 mg/m^2^ and LoDEX 20 mg (10 mg for patients aged >75 years). Secondary endpoints included safety, ORR (better than or equal to partial response (PR)), time to response (TTR) and duration of response (DOR).

Oral pomalidomide was administered on days 1–14 of a 21-day cycle. IV bortezomib was administered on days 1, 4, 8 and 11 for cycles 1–8 and then on days 1 and 8 for cycles ⩾9. Oral LoDEX was administered on days of and after bortezomib dosing. During the study, SC bortezomib became available with a favorable safety profile and low incidence of peripheral neuropathy. The study protocol was amended to include a cohort of patients who received SC bortezomib as part of the pomalidomide, bortezomib and LoDEX regimen at the MTD that was established with the IV formulation. After completing the first cycle of treatment, patients could continue the study at the assigned dose level until disease progression or unacceptable toxicity. Patients were evaluated every 21 days until and 28 days after treatment discontinuation.

In addition to study therapy, patients were required to receive thromboembolism prophylaxis (aspirin or low-molecular-weight heparin) and antiviral prophylaxis (for example, acyclovir) was recommended. Patients were allowed to receive red blood cell and platelet transfusions as needed and bisphosphonate therapy for myeloma-associated bone disease. The prophylactic use of hematopoietic growth factors was not allowed during cycle 1. Chronic use of steroids or other immunosuppressive therapies was not permitted. Pomalidomide dose interruptions and reductions per protocol were similar to those in the NIMBUS phase 3 trial and have been described previously.^10, 13, 20^ Dose modifications for bortezomib and dexamethasone were in accordance with their respective package inserts and institutional guidelines.^21^

The MTD was defined as the dose level preceding the dose level at which a dose-limiting toxicity (DLT) was observed in ⩾2 patients in the first 21-day cycle. Once the MTD was identified, an additional 6 patients were treated at the MTD to confirm safety and assess preliminary efficacy of pomalidomide, IV bortezomib and LoDEX. If a patient had a dose reduction of pomalidomide or bortezomib or discontinued treatment during cycle 1 for any reason other than a DLT, missed multiple doses of study drug, or received hematopoietic growth factors during cycle 1 before a DLT was declared, the patient was replaced within the given dose-escalation cohort for the purposes of determining the MTD.

A DLT was defined as any of the following toxicities occurring during the first cycle of treatment: grade 4 neutropenia (absolute neutrophil count of <500/μl) lasting for >7 days, febrile neutropenia (absolute neutrophil count of <1000/μl and temperature of >38.3 °C or a temperature of ⩾38.3 °C lasting for >1 h), grade 3 thrombocytopenia (platelet count of ⩾25 000 to <50 000/μl) with significant bleeding and need for hospitalization and/or platelet transfusion, grade 4 thrombocytopenia (platelet count of <25 000/μl) with a ⩾30% decrease in platelet count from baseline and requiring >1 platelet transfusion, grade 4 infection and grade ⩾3 toxicities related to pomalidomide (nausea, vomiting, constipation and/or diarrhea with optimal symptomatic treatment or fatigue lasting for >7 days).

### Toxicity and response assessments

Safety was routinely monitored per protocol frequency and included physical examinations, clinical laboratory evaluations, venous thromboembolism monitoring, electrocardiograms, second primary malignancy monitoring and adverse event (AE) monitoring. AEs were coded according to the Medical Dictionary for Regulatory Activities, version ⩾14. The severity of AEs and clinical laboratory values were graded according to the National Cancer Institute Common Terminology Criteria for Adverse Event (NCI CTCAE) version ⩾4.0, with the exception of rash, which was graded using NCI CTCAE version 3.0. If a patient experienced an AE on multiple occasions, the event was counted only once and by the greatest severity. Treatment-emergent AEs (TEAEs) were defined as any AEs occurring or worsening on or after the first dose of a study medication and within 28 days after the last dose.

Tumor response was assessed according to the International Myeloma Working Group criteria at every cycle on day 1 starting at cycle 2 and treatment discontinuation.^22^ Response assessments included measurement of myeloma paraprotein by protein electrophoresis and immunofixation; measurement of serum immunoglobulin, serum-free light chain and corrected serum calcium levels; bone marrow aspiration/biopsy; radiological imaging for lytic bone lesions; and clinical and/or radiological measurements for extramedullary plasmacytoma.

### Statistical analysis

Patient demographics, baseline disease characteristics, medical history and prior or concomitant medications were summarized using descriptive statistics or frequency tabulations.

The efficacy-evaluable population included all enrolled patients who received ⩾1 dose of study medication and who had measurable disease with serum (⩾0.5 g/dl) and/or urine protein (⩾200 mg/24 h) electrophoresis at baseline and at ⩾1 postbaseline assessment. The ORR (better than or equal to PR), together with the relative proportions in each response category, was summarized. For responders (better than or equal to PR), TTR, which is defined as the time from the first date of dosing to the first date of documented response, and DOR, which is defined as time from the earliest date of documented response to the earliest date when disease progression was confirmed, were also summarized.

The safety population included all patients who received ⩾1 dose of study medication. TEAEs, AEs leading to study medication discontinuation, AEs leading to dose reduction/interruption, AEs related to study medication, serious AEs and AEs leading to death were summarized by system organ class and preferred term for each treatment group. A summary of AEs with NCI CTCAE grade ⩾3, as well as the most frequent preferred terms, are provided. All deaths and reasons for death were summarized. Statistical analyses were conducted using SAS version ⩾9.1.

## Results

### Patients and treatments

Between March 2012 and August 2014, a total of 34 patients with RRMM were enrolled and treated. Twelve patients received treatment in the first 4 dose-escalation cohorts (3 patients at each dose level) and 3 patients were initially treated at the MPD (level 5; Figure 1). None of the first 15 patients treated experienced a DLT, and there was no need to replace any of the patients within the dose-escalation cohorts. Therefore, the MTD was defined as the MPD: pomalidomide 4 mg, bortezomib 1.3 mg/m^2^ and LoDEX 20 mg (10 mg for patients aged >75 years). The MTD cohort was subsequently expanded to include 7 more patients for a total of 10 patients. An additional cohort was added that comprised 12 patients treated with pomalidomide, LoDEX and SC bortezomib at the MTD.

The results for this study are presented according to 3 patient groupings: group 1 includes the 12 patients in the first 4 dose-escalation cohorts; group 2 includes 10 patients (3 patients initially treated at the MPD level and then expanded to include 7 more patients at the MPD level) at the MTD with IV bortezomib; and group 3 includes 12 patients at the MTD with SC bortezomib (Table 1).

Of the study patients, 59 were male and 56 and 44% had an Eastern Cooperative Oncology Group performance status of 0 and 1, respectively (Table 1). Median patient age was 58.5 years (range, 36–76 years). The median time from initial diagnosis was 3.4 years (range, 0.7–12.1 years); in the MTD groups with IV bortezomib and MTD groups with SC bortezomib, it was 4.2 years (range, 1.5–8.2 years) and 2.0 years (range, 0.7–9.1 years), respectively. All patients were refractory to lenalidomide, and all were exposed to a prior PI (97% bortezomib; 6% ixazomib). All patients had progressive disease after the last myeloma regimen, and the majority (91.2%) had progressed ⩽60 days after the last regimen. Twenty-three patients (68%) underwent prior stem cell transplant (SCT). The median number of prior lines of antimyeloma therapy received was 2 (range, 1–4). Compared with the IV bortezomib cohort, fewer patients in the SC bortezomib cohort received ⩾2 prior lines of antimyeloma treatment and prior transplant. At screening, 14 patients had peripheral sensory neuropathy, 11 of whom had a grade 1 event.

At the time of data cutoff (2 April 2015), all patients had discontinued study treatment (Table 2). The most common reason for treatment discontinuation was PD in 22 patients (65%): 9 (75%) in the dose-escalation cohort and 6 (60%) and 7 (58%) in the MTD cohorts with IV and SC bortezomib, respectively. One patient discontinued pomalidomide treatment due to metastatic pancreatic cancer diagnosed 1.1 months after study treatment initiation, and 3 patients discontinued because they went on to receive SCT. As of November 2016, 13 patients are in long-term follow-up.

### Treatment exposure and safety

The median duration of treatment was 6.2 months (range, 1.2–27.6 months), and the median number of treatment cycles received was 9 (range, 2–36) for all treated patients (Table 3). Patients in the MTD IV bortezomib cohort received a median of 11 (range, 2–19) and patients in the MTD SC bortezomib cohort received a median of 8 (range, 3–15) cycles of treatment. The median relative dose intensity was 0.9 for pomalidomide throughout the study and 0.9 for bortezomib during the first 8 cycles of treatment. The incidences of dose interruptions and reductions due to TEAEs were 79 and 38% for pomalidomide and 74 and 44% for bortezomib, respectively. The number of dose interruptions was similar regardless of method of bortezomib administration. However, the number of reductions of pomalidomide and bortezomib dose due to TEAEs was slightly less frequent in the IV (40 and 30%, respectively) vs SC (50 and 58%, respectively) bortezomib cohort. Neutropenia was the most common TEAE leading to pomalidomide dose reductions (9%). Infections (47%), neutropenia (26%), pneumonia (9%), peripheral sensory neuropathy (9%), dizziness (9%) and fatigue (9%) were common TEAEs (⩾3 patients) leading to pomalidomide dose interruptions. Peripheral sensory neuropathy (12%) was the most common TEAE (⩾3 patients) leading to bortezomib dose reductions, whereas interruptions were primarily due to infections (44%), neutropenia (21%), pneumonia (12%) and fatigue (9%).

All of the 34 patients included in the safety population had ⩾1 TEAE; 29 (85%) had ⩾1 grade 3/4 TEAE (Table 4). Neutropenia and thrombocytopenia were the most common grade 3/4 TEAEs. The incidence of these events was higher in the IV bortezomib cohort (80 and 40%, respectively) compared with the SC bortezomib cohort (25 and 17%, respectively). Eighteen patients (53%) had treatment-emergent peripheral neuropathy, with 6 out of 12 (50%) in the escalation cohort, 3 out of 10 (30%) in the IV bortezomib cohort and 9 out of 12 (75%) in the SC bortezomib cohort; none of the events were grade 3/4. Treatment-emergent deep vein thrombosis was uncommon (6%); no patients experienced a grade 3/4 event. One death occurred during treatment cycle 3 as a result of cardiac arrest unrelated to study drugs.

### Efficacy

**Figure 2:**
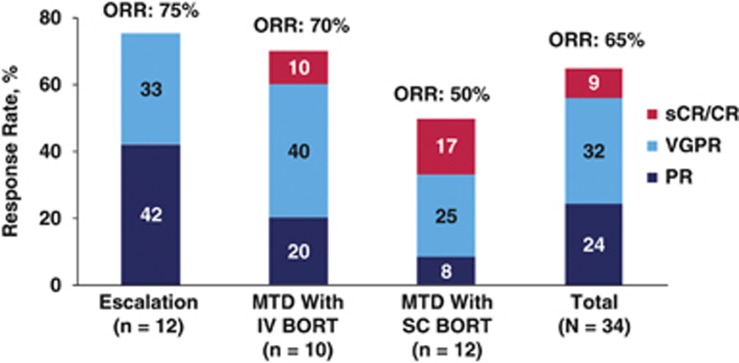
Response outcomes. All treated patients were evaluated for response (better than or equal to PR). TTR and DOR were reported for patients who achieved response. BORT, bortezomib; CR, complete response; sCR, stringent complete response; VGPR, very good PR.

All 34 patients were evaluable for tumor response (Figure 2). Disease control with at least stable disease was reported for all patients. The ORR (better than or equal to PR) for all treated patients was 65%. One patient in the MTD IV bortezomib cohort had a stringent complete response (sCR) and 2 patients in the MTD SC bortezomib cohort had a CR. Patients with sCR/CR received 1 or 2 prior lines of therapy (Table 5). ORR was 59% for all patients treated at the MTD: 70 and 50% in the IV and SC bortezomib cohorts, respectively. For all responding patients (*n*=22), the median TTR was 1.0 months (range, 0.7–5.1 months) and the median DOR was 7.4 months (95% CI, 4.4–9.6 months). This was similar for the patients treated at the MTD: median TTR of 0.9 months (range, 0.7–3.1 months) and median DOR of 7.4 months (95% CI, 4.1-not estimable).

## Discussion

Preclinical and clinical studies have proven the clinical benefit of combination therapy with an immunomodulatory agent and a PI for the treatment of patients with RRMM.^4, 5, 6, 7^ In this phase 1 trial, we demonstrated that triple therapy with pomalidomide, bortezomib and LoDEX was highly active, resulting in an ORR of 65% and disease control in 100%, despite all patients being refractory to lenalidomide and nearly all having prior exposure to bortezomib. Responses were durable, lasting a median of 7.4 months. The MTD of the combination regimen was established at the MPD of pomalidomide 4 mg, IV or SC bortezomib 1.3 mg/m^2^ and LoDEX 20 mg (10 mg for patients aged >75 years). At this therapeutic dose level, 3 patients achieved sCR/CR.

The regimen of pomalidomide, bortezomib and LoDEX was well tolerated, and toxicities proved manageable, regardless of mode of bortezomib administration. Patients were able to receive a median of 9 cycles of therapy, and no patients discontinued treatment due to a treatment-related event. The incidences of dose interruptions and reductions for pomalidomide and bortezomib were consistent with those of prior studies.^13, 19^ There were also no reports of grade 3/4 peripheral neuropathy, deep vein thrombosis or other toxicities sometimes associated with immunomodulatory agents and/or PIs.^10, 21, 23, 24^ Consistent with the findings of the SC vs IV bortezomib phase 3 noninferiority trial, grade 3/4 TEAEs were less frequent with SC vs IV bortezomib administration.^19^ However, this finding may have been influenced by fewer patients receiving ⩾2 prior lines of antimyeloma treatment and prior transplant in the SC vs IV bortezomib cohorts. Patients in the SC cohort also received fewer cycles of treatment, which may have influenced the incidence of TEAEs. The finding that patients received fewer cycles of SC vs IV bortezomib may be due to the timing of when patients discontinued treatment for SCT; 1 patient in the IV group and 2 patients in the SC group went on to receive SCTs.

In studies of pomalidomide plus LoDEX, approximately one-third of patients with advanced RRMM achieved a tumor response (better than or equal to PR).^25^ As demonstrated in this trial and others, the addition of PIs to IMiD combinations has the potential to lead to deeper and more durable responses.^26, 27, 28, 29, 30^ Preliminary results of a phase 1/2 trial of once-weekly bortezomib with pomalidomide and LoDEX demonstrated a response rate of 85, with 19 and 45% of patients achieving sCR/CR and at least very good PR, respectively.^29^ Responses were durable, lasting a median of 13.7 months. This patient population differed somewhat because <30% of patients had refractory disease and just over one-half had prior exposure to bortezomib. In another phase 1 trial with the PI carfilzomib, given in combination with pomalidomide and LoDEX, the ORR was 50%, with a clinical benefit rate of 66%.^28^ Similar to data from the phase 1 trial presented here, all patients were refractory to lenalidomide, although the patients were more heavily pretreated (median 6 prior regimens (range, 2–12)) and nearly all were refractory to bortezomib. Results of the phase 1 portion of the Alliance A061202 study of an oral PI, ixazomib, given in combination with pomalidomide and LoDEX, demonstrated an ORR of 55% in patients with RRMM who had received a median of 3 prior lines of therapy (range, 2–10) and who were refractory to lenalidomide and a PI.^26^

Pomalidomide-based triple therapy combination regimens with other drug classes have also demonstrated high antimyeloma activity in patients with RRMM, including the triple therapy regimens of pomalidomide plus dexamethasone in combination with cyclophosphamide (ORR, 65–67%),^31, 32^ the monoclonal antibodies anti-CD38 (daratumumab; ORR, 71%)^33^ and anti–programmed cell death protein 1 (pembrolizumab; ORR, 60%)^34^ and a histone deacetylase inhibitor (ACY-241; ORR, 46%).^35^ Taken together, these studies demonstrate the utility of pomalidomide and dexamethasone as a platform for combining novel agents.

In conclusion, the MTD of pomalidomide 4 mg, bortezomib 1.3 mg/m^2^ and LoDEX 20 mg (10 mg for patients aged >75 years) was well tolerated and highly active in patients with RRMM who were refractory to lenalidomide and had been previously exposed to bortezomib. These findings support further evaluation in clinical trials and suggest that pomalidomide, bortezomib and LoDEX may be an important new treatment option for patients with RRMM. A large randomized, multicenter, international, phase 3 trial, MM-007 (OPTIMISMM), to confirm these findings is currently ongoing and is close to completing enrollment (ClinicalTrials.gov NCT01734928).

Future directions include the addition of other novel agents to this platform, such as the monoclonal antibodies discussed previously, as well as histone deacetylase inhibitors and other promising next-generation small molecules.^36, 37, 38^

## Figures and Tables

**Table 1:** Baseline patient demographic, disease and treatment characteristics

	*Escalation (*n=*12)*	*MTD with IV bortezomib (*n=*10)*	*MTD with SC bortezomib (*n=*12)*	*Total (*N=*34)*
Median age, y (range)	57.5 (36–75)	58.5 (49–67)	61 (43–76)	58.5 (36–76)
Male, *n* (%)	6 (50)	6 (60)	8 (67)	20 (59)
				
*ECOG performance status*, n *(%)*				
0	5 (42)	6 (60)	8 (67)	19 (56)
1	7 (58)	4 (40)	4 (33)	15 (44)
				
*ISS stage*, n *(%)*^a^				
I	1 (8)	5 (50)	6 (50)	12 (35)
II	1 (8)	1 (10)	5 (42)	7 (21)
III	3 (25)	1 (10)	0	4 (12)
Missing	7 (58)	3 (30)	1 (8)	11 (32)
				
*β*_2_*-microglobulin (mg/l)*				
Median (range)	2.4 (1.3–4.4)	2.3 (1.6–6.7)	2.6 (1.4–6.5)	2.5 (1.3–6.7)
				
*Hemoglobin (g/l)*				
Median (range)	113.5 (87.0–148.0)	108.5 (83.0–137.0)	123.0 (90.0–154.0)	115.0 (83.0–154.0)
				
*Platelets, × 10^9^/L*				
Median (range)	150 (56.0–254.0)	169.0 (59.0–304.0)	191.5 (96.0–298.0)	165.0 (56.0–304.0)
				
*ANC, × 10^9^/L*				
Median (range)	2.2 (1.1–3.5)	2.0 (1.2–5.4)	3.0 (0.9–6.7)	2.2 (0.9–6.7)
				
*Presence of bone lesions*				
Yes/no, *n* (%)	9 (75)/3 (25)	6 (60.0)/4 (40.0)	9 (75.0)/3 (25)	24 (70.6)/10 (29.4)
Median prior lines of treatment (range)	2 (1–4)	2 (1–3)	1 (1–4)	2 (1–4)
⩾2 prior lines of treatment, *n* (%)	8 (67)	7 ((70)	4 (33)	19 (56)
				
*Time from initial diagnosis (y)*				
Median (range)	4.0 (2.4–12.1)	4.2 (1.5–8.2)	2.0 (0.7–9.1)	3.4 (0.7–12.1)
Prior lenalidomide and PI, *n* (%)	12 (100)	10 (100)	12 (100)	34 (100)
Prior dexamethasone, *n* (%)	12 (100)	10 (100)	12 (100)	34 (100)
Prior thalidomide, *n* (%)	6 (50)	3 (30)	3 (25)	12 (35)
Prior bortezomib, *n* (%)	12 (100)	10 (100)	11 (92)	33 (97)
Prior ixazomib, *n* (%)	0	1 (10)	1 (8)	2 (6)
Prior SCT, *n* (%)	12 (100)	8 (80)	3 (25)	23 (68)

Abbreviations: ANC, absolute neutrophil count; ECOG, Eastern Cooperative Oncology Group; ISS, International Staging System; IV, intravenous; MTD, maximum tolerated dose; PI, proteasome inhibitor; SC, subcutaneous; SCT, stem cell transplant.

a Percentage of patients with data available.

**Table 2:** Patient disposition

	*Escalation (*n=*12)*	*MTD with IV bortezomib (*n=*10)*	*MTD with SC bortezomib (*n=*12)*	*Total (*N=*34)*
On treatment, *n* (%)	0	0	0	0
Discontinued, *n* (%)	12 (100)	10 (100)	12 (100)	34 (100)
PD	9 (75)	6 (60)	7 (58)	22 (65)
Consent withdrawn	2 (17)	1 (10)	0	3 (9)
AE	0	1 (10)^a^	0	1 (3)
Death	0	0	1 (8)^b^	1 (3)
Lost to follow-up	0	0	1 (8)	1 (3)
Other	1 (8)^c^	2 (20)^d^	3 (25)^e^	6 (18)

Abbreviations: AE, adverse event; IV, intravenous; MTD, maximum tolerated dose; PD, progressive disease; SC, subcutaneous.

a One patient discontinued study treatment in cycle 2 due to metastatic pancreatic cancer unrelated to treatment.

b One patient died due to cardiac arrest unrelated to treatment in cycle 3.

c One patient was unable to switch to SC bortezomib.

d One patient proceeded to transplant and 1 patient discontinued for noncompliance.

e Two patients proceeded to transplant and 1 patient discontinued due to clinical progression.

**Table 3:** Treatment exposure, dose interruptions and dose reductions

	*Escalation (*n=*12)*	*MTD with IV bortezomib (*n=*10)*	*MTD with SC bortezomib (*n=*12)*	*Total (*N=*34)*
*Median duration of treatment, mo (range)*				
Overall	6.3 (2.8–27.6)	7.6 (1.2–18.5)	6.0 (2.1–10.6)	6.2 (1.2–27.6)
Pomalidomide	6.1 (2.7–27.6)	7.6 (1.2–18.5)	6.0 (1.4–10.6)	6.2 (1.2–27.6)
Bortezomib	5.1 (2.8–27.6)	7.6 (1.2–18.5)	5.8 (2.1–10.6)	6.1 (1.2–27.6)
Dexamethasone	5.8 (2.8–27.6)	7.6 (1.2–18.5)	5.3 (0.7–10.6)	5.9 (0.7–27.6)
				
*Median relative dose intensity (range)*				
Pomalidomide	0.9 (0.6–1.0)	0.9 (0.6–1.1)	0.8 (0.2–1.0)	0.9 (0.2–1.1)
Bortezomib^a^	0.9 (0.5–1.1)	0.9 (0.6–1.0)	0.8 (0.4–1.0)	0.9 (0.4–1.1)
Dexamethasone^a^	0.7 (0.4–1.0)	0.8 (0.5–1.1)	0.5 (0.3–1.0)	0.6 (0.3–1.1)
				
*Interruption due to TEAE*, n *(%)*				
Pomalidomide	7 (58)	9 (90)	11 (92)	27 (79)
Bortezomib	7 (58)	8 (80)	10 (83)	25 (74)
Dexamethasone	8 (67)	8 (80)	11 (92)	27 (79)
				
*Reduction due to TEAE*, n *(%)*				
Pomalidomide	3 (25)	4 (40)	6 (50)	13 (38)
Bortezomib	5 (42)	3 (30)	7 (58)	15 (44)
Dexamethasone	9 (75)	7 (70)	8 (67)	24 (71)

Abbreviations: IV, intravenous; MTD, maximum tolerated dose; SC, subcutaneous; TEAE, treatment-emergent adverse event.

a During the first 8 cycles.

**Table 4:** Grade 3/4 TEAEs occurring in ⩾5% of all patients

*Grade 3/4 TEAE*, n *(%)*	*Escalation (*n=*12)*	*MTD with IV bortezomib (*n=*10)*	*MTD with SC bortezomib (*n=*12)*	*Total (*N=*34)*
Any	11 (92)	9 (90)	9 (75)	29 (85)
Neutropenia^a^	4 (33)	8 (80)	3 (25)^b^	15 (44)
Thrombocytopenia^c^	3 (25)	4 (40)	2 (17)	9 (26)
Pneumonia	2 (17)	3 (30)	1 (8)	6 (18)
Hypophosphatemia	3 (25)	1 (10)	1 (8)	5 (15)
Blood CPK increase	2 (17)	0	1 (8)	3 (9)
Anemia	1 (8)	0	1 (8)	2 (6)
Fatigue	0	0	2 (17)	2 (6)
Hyperglycemia	1 (8)	1 (10)	0	2 (6)
Hypokalemia	0	2 (20)	0	2 (6)
Dizziness	1 (8)	0	1 (8)	2 (6)
Tremor	1 (8)	1 (10)	0	2 (6)

Abbreviations: CPK, creatinine phosphokinase; IV, intravenous; MTD, maximum tolerated dose; SC, subcutaneous; TEAE, treatment-emergent adverse event.

a Neutropenia includes neutrophil count decreases.

b One patient experienced febrile neutropenia.

c Thrombocytopenia includes platelet count decreases.

**Table 5:** Response outcomes by number of prior lines of therapy

*Prior lines of antimyeloma therapy*	*Escalation (*n=*12)*		*MTD with IV bortezomib (*n=*10)*		*MTD with SC bortezomib (*n=*12)*	
	*1 (*n=*4)*	*⩾2 (*n=*8)*	*1 (*n=*3)*	*⩾2 (*n=*7)*	*1 (*n=*8)*	*⩾2 (*n=*4)*
ORR	3 (75.0)	6 (75.0)	2 (66.7)	5 (71.4)	3 (37.5)	3 (75.0)
sCR	0	0	0	1	0	0
CR	0	0	0	0	1	1
VGPR	1	3	1	3	2	1
PR	2	3	1	1	0	1
SD	1	2	1	2	5	1
Progressed/died after achieving response	2	6	2	2	1	1

Abbreviations: CR, complete response; IV, intravenous; MTD, maximum tolerated dose; ORR, overall response rate; PR, partial response; SC, subcutaneous; sCR, stringent complete response; SD, stable disease; VGPR, very good PR.

## References

[bib1] Cornell RF, Kassim AA. Evolving paradigms in the treatment of relapsed/refractory multiple myeloma: increased options and increased complexity. Bone Marrow Transplant 2016; 51: 479–491. 26726946 10.1038/bmt.2015.307 PMC4827007

[bib2] Ria R, Reale A, Vacca A. Novel agents and new therapeutic approaches for treatment of multiple myeloma. World J Methodol 2014; 4: 73–90. 25332907 10.5662/wjm.v4.i2.73 PMC4202483

[bib3] Kumar SK, Therneau TM, Gertz MA, Lacy MQ, Dispenzieri A, Rajkumar SV et al. Clinical course of patients with relapsed multiple myeloma. Mayo Clin Proc 2004; 79: 867–874. 15244382 10.4065/79.7.867

[bib4] Hideshima T, Chauhan D, Shima Y, Raje N, Davies FE, Tai Y et al. Thalidomide and its analogs overcome drug resistance of human multiple myeloma cells to conventional therapy. Blood 2000; 96: 2943–2950. 11049970

[bib5] Mitsiades N, Mitsiades CS, Poulaki V, Chauhan D, Richardson PG, Hideshima T et al. Apoptotic signaling induced by immunomodulatory thalidomide analogs in human multiple myeloma cells: therapeutic implications. Blood 2002; 99: 4525–4530. 12036884 10.1182/blood.v99.12.4525

[bib6] Richardson PG, Weller E, Lonial S, Jakubowiak AJ, Jagannath S, Raje NS et al. Lenalidomide, bortezomib, and dexamethasone combination therapy in patients with newly diagnosed multiple myeloma. Blood 2010; 116: 679–686. 20385792 10.1182/blood-2010-02-268862 PMC3324254

[bib7] Richardson PG, Xie W, Jagannath S, Jakubowiak A, Lonial S, Raje NS et al. A phase 2 trial of lenalidomide, bortezomib, and dexamethasone in patients with relapsed and relapsed/refractory myeloma. Blood 2014; 123: 1461–1469. 24429336 10.1182/blood-2013-07-517276 PMC4123434

[bib8] Durie BGM, Hoering A, Abidi MH, Rajkumar SV, Epstein J, Kahanic SP et al. Bortezomib, lenalidomide and dexamethasone vs. lenalidomide and dexamethasone induction followed by lenalidomide and dexamethasone maintenance in patients with newly diagnosed myeloma without intent for immediate autologous stem cell transplant: results of the randomised phase III SWOG trial S0777. Lancet 2016; 389: 519–527. 28017406 10.1016/S0140-6736(16)31594-X PMC5546834

[bib9] Quach H, Ritchie D, Stewart AK, Neeson P, Harrison S, Smyth MJ et al. Mechanism of action of immunomodulatory drugs (IMiDs) in multiple myeloma. Leukemia 2010; 24: 22–32. 19907437 10.1038/leu.2009.236 PMC3922408

[bib10] Pomalyst (pomalidomide) [package insert]. Celgene Corporation; Summit, NJ, USA, 2016.

[bib11] Leleu X, Attal M, Arnulf B, Moreau P, Traulle C, Marit G et al. Pomalidomide plus low dose dexamethasone is active and well tolerated in bortezomib and lenalidomide-refractory multiple myeloma: Intergroupe Francophone du Myélome 2009-02. Blood 2013; 121: 1968–1975. 23319574 10.1182/blood-2012-09-452375

[bib12] Richardson PG, Siegel DS, Vij R, Hofmeister CC, Baz R, Jagannath S et al. Pomalidomide alone or in combination with low-dose dexamethasone in relapsed and refractory multiple myeloma: a randomized phase 2 study. Blood 2014; 123: 1826–1832. 24421329 10.1182/blood-2013-11-538835 PMC3962162

[bib13] San Miguel J, Weisel K, Moreau P, Lacy M, Song K, Delforge M et al. Pomalidomide plus low-dose dexamethasone versus high-dose dexamethasone alone for patients with relapsed and refractory multiple myeloma (MM-003): a randomised, open-label, phase 3 trial. Lancet Oncol 2013; 14: 1055–1066. 24007748 10.1016/S1470-2045(13)70380-2

[bib14] Palumbo A, Dimopolous MA, Richardson PG, Siegel DS, Cavo M, Corradini P et al. A pooled analysis of the impact of age on outcomes in patients with refractory or relapsed and refractory multiple myeloma treated with pomalidomide plus low-dose dexamethasone. The 21st Congress of the European Hematology Association: Copenhagen, Denmark, 9–12, 2016; Poster of abstract E1295.

[bib15] Leleu X, Karlin L, Macro M, Hulin C, Garderet L, Roussel M et al. Pomalidomide plus low-dose dexamethasone in multiple myeloma with deletion 17p and/or translocation (4;14): IFM 2010-02 trial results. Blood 2015; 125: 1411–1417. 25575538 10.1182/blood-2014-11-612069

[bib16] Dimopoulos MA, Weisel KC, Song KW, Delforge M, Karlin L, Goldschmidt H et al. Cytogenetics and long-term survival of pomalidomide and low-dose dexamethasone in refractory or relapsed and refractory multiple myeloma. Haematologica 2015; 100: 1327–1333. 26250580 10.3324/haematol.2014.117077 PMC4591765

[bib17] Weisel KC, Dimopoulos MA, Moreau P, Lacy MQ, Song KW, Delforge M et al. Analysis of renal impairment in MM-003, a phase 3 study of pomalidomide + low-dose dexamethasone vs high-dose dexamethasone in refractory or relapsed and refractory multiple myeloma. Haematologica 2016; 101: 872–878. 27081177 10.3324/haematol.2015.137083 PMC5004467

[bib18] Siegel DS, Weisel KC, Dimopoulos MA, Baz R, Richardson P, Delforge M et al. Pomalidomide plus low-dose dexamethasone in patients with relapsed/refractory multiple myeloma and moderate renal impairment: a pooled analysis of three clinical trials. Leuk Lymphoma 2016; 57: 2833–2838. 27267105 10.1080/10428194.2016.1177181

[bib19] Moreau P, Pylypenko H, Grosicki S, Karamanesht I, Leleu X, Grishunina M et al. Subcutaneous versus intravenous administration of bortezomib in patients with relapsed multiple myeloma: a randomised, phase 3, non-inferiority study. Lancet Oncol 2011; 12: 431–440. 21507715 10.1016/S1470-2045(11)70081-X

[bib20] Imnovid (pomalidomide) [summary of product characteristics]. Celgene Europe; Uxbridge, UK, 2016.

[bib21] Velcade (bortezomib) for injection [package insert]. Millennium Pharmaceuticals; Cambridge, MA, USA, 2015.

[bib22] Durie BG, Harousseau JL, Miguel JS, Blade J, Barlogie B, Anderson K et al. International uniform response criteria for multiple myeloma. Leukemia 2006; 20: 1467–1473. 16855634 10.1038/sj.leu.2404284

[bib23] Revlimid (lenalidomide) [package insert]. Celgene Corporation; Summit, NJ, USA, 2015.

[bib24] Thalomid (thalidomide) [package insert]. Celgene Corporation; Summit, NJ, USA, 2015.

[bib25] Zou Y, Ma X, Yu H, Hu C, Fan L, Ran X. Carfilzomib/pomalidomide single-agent or in combination with other agents for the management of relapsed/refractory multiple myeloma : a meta-analysis of 37 trials. Oncotarget 2017; 8: 39805–39817. 27458170 10.18632/oncotarget.10768 PMC5503655

[bib26] Voorhees PM, Mulkey F, Hassoun H, Paba-Prada CE, Efebera YA, Hoke E et al. Alliance A061202. A phase I/II study of pomalidomide, dexamethasone and ixazomib versus pomalidomide and dexamethasone for patients with multiple myeloma refractory to lenalidomide and proteasome inhibitor based therapy: phase I results. Blood 2015; 126: Abstract 375.

[bib27] Shah J, Niesvizky R, Stadtmauer E, Rifkin RM, Berenson J, Berdeja JG et al. Oprozomib, pomalidomide, and dexamethasone (OPomd) in patients (pts) with relapsed and/or refractory multiple myeloma (RRMM): initial results of a phase 1b study (NCT01999335). Blood 2015; 126: Abstract 378.

[bib28] Shah JJ, Stadtmauer EA, Abonour R, Cohen AD, Bensinger WI, Gasparetto C et al. Carfilzomib, pomalidomide, and dexamethasone for relapsed or refractory myeloma. Blood 2015; 126: 2284–2290. 26384354 10.1182/blood-2015-05-643320 PMC4643003

[bib29] Lacy MQ, LaPlant BR, Laumann KM, Kumar S, Gertz MA, Hayman SR et al. Pomalidomide, bortezomib and dexamethasone (PVD) for patients with relapsed lenalidomide refractory multiple myeloma (MM). Blood 2014; 124: Abstract 304. 10.1182/blood-2017-05-782961 PMC5606008 28684537

[bib30] Krishnan A, Kapoor P, Palmer J, Tsai N, Kumar S, Lonial S et al. A phase I/II study of ixazomib (Ix) pomalidomide (POM) dexamethasone (DEX) in relapsed refractory (R/R) multiple myeloma: initial results. J Clin Oncol 2016; 34: Abstract 8008.

[bib31] Baz RC, Martin TG 3rd, Lin HY, Zhao X, Shain KH, Cho HJ et al. Randomized multicenter phase II study of pomalidomide, cyclophosphamide, and dexamethasone in relapsed refractory myeloma. Blood 2016; 127: 2561–2568. 26932802 10.1182/blood-2015-11-682518

[bib32] Chari A, Cho HJ, Lau K, Morgan G, Florendo E, Catamero D et al. A phase II study of pomalidomide, daily low dose oral cyclophosphamide, and dexamethasone in relapsed/refractory multiple myeloma. American Society of Hematology 58th Annual Meeting and Exposition: San Diego, CA, USA, December 3–62016; Poster of abstract 4520.

[bib33] Chari A, Lonial S, Suvannasankha A, Fay JW, Arnulf B, Ifthikharuddin JJ et al. Open-label, multicenter, phase 1b study of daratumumab in combination with pomalidomide and dexamethasone in patients with at least 2 lines of prior therapy and relapsed or relapsed and refractory multiple myeloma. Blood 2015; 126: Abstract 508.

[bib34] Badros AZ, Kocoglu MH, Ma N, Rapoport AP, Lederer E, Philip S et al. A phase II study of anti PD-1 antibody pembrolizumab, pomalidomide and dexamethasone in patients with relapsed/refractory multiple myeloma (RRMM). Blood 2015; 126: Abstract 506.

[bib35] Niesvizky R, Richardson PG, Yee AJ, Nooka AK, Raab MS, Shain KH et al. Selective HDAC6 inhibitor ACY-241, and oral tablet, combined with pomalidomide and dexamethasone: safety and efficacy of escalation and expansion cohorts in patients with relapsed or relapsed-and-refractory multiple myeloma (ACE-MM-200 study). American Society of Hematology 58th Annual Meeting and Exposition: San Diego, CA, USA, December 3–62016; Poster of abstract 3307.

[bib36] Kaufman JL, Fabre C, Lonial S, Richardson PG. Histone deacetylase inhibitors in multiple myeloma: rationale and evidence for their use in combination therapy. Clin Lymphoma Myeloma Leuk 2013; 13: 370–376. 23787122 10.1016/j.clml.2013.03.016

[bib37] Laubach JP, Voorhees PM, Hassoun H, Jakubowiak A, Lonial S, Richardson PG. Current strategies for treatment of relapsed/refractory multiple myeloma. Expert Rev Hematol 2014; 7: 97–111. 24471924 10.1586/17474086.2014.882764

[bib38] Laubach JP, Paba Prada CE, Richardson PG, Longo DL. Daratumumab, elotuzumab, and the development of therapeutic monoclonal antibodies in multiple myeloma. Clin Pharmacol Ther 2017; 101: 81–88. 27806428 10.1002/cpt.550

