# Correction: Pomalidomide, bortezomib, and low-dose dexamethasone in lenalidomide-refractory and proteasome inhibitor-exposed myeloma

**DOI:** 10.1038/s41375-018-0235-5

**Published:** 2018-09-14

**Authors:** P. G. Richardson, C. C. Hofmeister, N. S. Raje, D. S. Siegel, S. Lonial, J. Laubach, Y. A. Efebera, D. H. Vesole, A. K. Nooka, J. Rosenblatt, D. Doss, M. H. Zaki, A. Bensmaine, J. Herring, Y. Li, L. Watkins, M. S. Chen, K. C. Anderson

**Affiliations:** 1 Jerome Lipper Multiple Myeloma Center, Department of Medical Oncology, Dana-Farber Cancer Institute, Harvard Medical School, Boston, MA USA; 2 Division of Hematology, Department of Internal Medicine, The Ohio State University, Columbus, OH USA; 3 Massachusetts General Hospital, Boston, MA USA; 4 John Theurer Cancer Center, Hackensack University Medical Center, Hackensack, NJ USA; 5 Department of Hematology and Medical Oncology, Winship Cancer Institute, Emory University School of Medicine, Atlanta, GA USA; 6 Beth Israel Deaconess Medical Center, Harvard Medical School, Boston, MA USA; 7 Celgene Corporation, Summit, NJ USA

Correction to: *Leukemia* ; 10.1038/leu.2017.173; published online 2 June 2017

Following the publication of this article, the authors noted that the pomalidomide dose for the additional SC cohort in Fig.1 was incorrectly listed. The correct dose for pomalidomide in the additional SC cohort should be the maximum tolerated dose of 4 mg/day, not 2 mg/day as listed in the original Fig. 1. The authors apologize for any inconvenience caused.

